# Tuning Biodegradation and Physicochemical Features of PLA/HAp Biomaterials by Incorporating Nanofibrillated Cellulose Through a Colloidal Processing Route

**DOI:** 10.3390/polym17121595

**Published:** 2025-06-07

**Authors:** Maria Eugenia Juan-Cano, Zoilo Gonzalez, Esther Rincón, Antonio Javier Sanchez-Herencia, Begoña Ferrari, Ana Ferrández-Montero

**Affiliations:** 1 Instituto de Cerámica y Vidrio (ICV), Consejo Superior de Investigaciones Científicas (CSIC), 28049 Madrid, Spain; eugeniajuancano@gmail.com (M.E.J.-C.); ajsanchez@icv.csic.es (A.J.S.-H.); bferrari@icv.csic.es (B.F.); 2 BioPren Group (RNM940), Chemical Engineering Department, Instituto Químico Para la Energía y el Medioambiente (IQUEMA), Faculty of Science, Universidad de Córdoba (UCO), 14014 Córdoba, Spain; zgonzalez@uco.es (Z.G.); b32rirue@uco.es (E.R.); 3 Unidad Asociada CSIC-UCO, Fabricación Aditiva de Materiales Compuestos Basados en Celulosa Funcionalizada, Obtenida de Residuos de Biomasa, 14014 Córdoba, Spain

**Keywords:** polylactic acid, hydroxyapatite, nanofibrillated cellulose, porosity modification, biodegradability

## Abstract

Biomaterials play a fundamental role in providing a porous structure that mimics the natural structure of human bone and serves as a support while tissue regenerates. With the use of biodegradable materials, it is possible to avoid unnecessary second surgeries for implant removal. The main objective of this article has been focused on modifying the degradation rate of a biodegradable composite material based on polylactic acid (PLA) reinforced with hydroxyapatite (HAp) by incorporating nanofibrillated cellulose (NFC), capable of tuning the porosity within the matrix. To introduce NFC into the composite material, a colloidal processing approach was chosen to improve and ensure its compatibility with the polymeric matrix. The incorporation of different ratios of NFC generally decreases the mechanical properties, but by adjusting the ratio of HAp/NFC content, this parameter is normalized. The hydrophilicity of the composite is improved by HAp/NFC incorporation, and degradation tests confirm that an increase in the percentage of NFC in the matrix is directly proportional to an increase in the degradation rates of the material. These results represent a significant improvement in personalized medicine, where the design of biodegradable biomaterials with hierarchical and controlled porosity opens new paths in the development of therapies and treatments personalized for each patient.

## 1. Introduction

Over the past few decades, bone tissue engineering has emerged as a critical approach for addressing bone injuries, providing an alternative to autografts and allografts by combining biomaterials and cells to stimulate cellular regeneration in a more efficient and lower-risk manner [1]. Artificial bone grafts make use of biocompatible materials to craft three-dimensional porous structures, known as scaffolds, which act as temporary support for damaged bone and cellular structures. Gradually degrading, these scaffolds encourage osteocyte proliferation and differentiation, ultimately leading to tissue regeneration and the scaffold’s replacement [2]. Successful tissue growth within the implant during recovery is essential to achieve a uniform load distribution and the scaffold’s integration with the natural tissue [3,4]. The ideal biomaterial combination for bone regeneration involves a combination of polymeric and ceramic materials that can emulate natural bone properties and composition [5]. However, the fabrication of such materials faces challenges, particularly the difficulty of uniformly dispersing the inorganic phase of the bioceramic within the organic polymeric matrix. The poor compatibility and the interfacial bonding forces between these materials can lead to the shrinkage and deformation of the polymeric matrix in contact with the ceramic surface, potentially causing microcracks and compromising the material’s mechanical properties [2].

Hydroxyapatite (HAp), a ceramic biomaterial, mimics the mineral composition and structure of vertebrate’s teeth and bones. Its bioactivity, biocompatibility, and processability make it an attractive option for implantable materials [6,7]. HAp contains calcium and phosphorus, metabolizable by the human body, and hydroxyl groups that interact with human tissues, allowing for potential absorption and replacement by the patient’s bone tissue. It can be used as solid scaffolds with varying porosities, facilitating cell adhesion and bone formation, or as a coating to enhance implant integration, such as with titanium implants. Although inherently brittle, combining HAp with polymers, both natural and synthetic, can yield composite materials with improved properties [8]. Polylactic acid (PLA), a thermoplastic polymer derived from natural sources, offers properties like high tensile strength, biodegradability, biocompatibility, and simple processing. However, it suffers from low ductility, poor thermal properties, and slow crystallization [9]. Current interests are focused on modifying PLA to enhance its bioactivity, especially for bone regeneration. Efforts involve combining it with components like HAp to promote the adhesion of new tissues. This combination can lead to mechanical properties analogous to natural bone, as demonstrated by Zhang et al. in their 3D-printed PLA/HAp scaffold research, in which a material containing 50% nHA exhibited a compressive strength of 14 MPa, similar to the higher reference value of cancellous bone [10]. PLA and HAp composite materials also show compatibility with cells, including fibroblasts and osteoblast precursors, due to an increased cell number and cellular adhesion [11].

Recent advances have demonstrated that the incorporation of other types of fillers, such as biopolymers in PLA composites, also enhances some of the drawbacks of PLA [12]. Attending to their intrinsic mechanical properties, the capability of nanocellulose or nanofibrillated cellulose (NFC) as a filler or support of other phases is an emerging alternative to proper PLA composites with added value [13]. For the biomedical sector, cellulose and cellulose derivatives-based materials are biocompatible, biodegradable, and environmentally friendly compounds that offer customizable porosity, mechanical strength enhancement, and a high surface-to-volume ratio when combined with other polymers [14]. The medical community has begun using NFC for cellular scaffolds and drug delivery due to its water content, chemical versatility, unique properties, such as low density, abundance, high-functionalization possibilities, and its vast applicability [15,16]. Nanofibers mimic the extracellular matrix, promoting cell adhesion and differentiation [17]. Additionally, nanocellulose is able to generate heterogeneous porosity in the PLA matrix, which, in turn, could modulate the material biodegradation profile [18]. Nevertheless, other issues arise during the mixing process in the compatibility of cellulose and the polymer matrix due to the hydrophilicity of cellulose and the hydrophobicity of the polymer matrix, in which cellulose requires surface modification to improve the compatibility and dispersion of cellulose in the polymer matrix. One example is that, prior to obtaining NFC, cellulose fibers can be subjected to various chemical pretreatments that not only facilitate the delamination process to obtain NFC but also modify their surface to overcome these incompatibilities. In this sense, the oxidation of cellulose fibers with sodium hypochlorite (NaClO) using the 2,2,6,6-tetramethyl-1-piperidinyloxy free radical as a catalyst for the reaction (chemical pretreatment commonly known as TEMPO-mediated oxidation) addresses dispersion concerns by exchanging the -OH groups for new carboxylic acid and aldehyde groups on the cellulose surface [19,20].

This study aimed to create a new biomaterial with controllable biodegradation rates by combining NFC with a composite of HAp and PLA. The initial step was the modification of the cellulose nanofibers’ surface to ensure their integration into the hydrophobic PLA matrix using HA, creating heterostructures of HAp/NFC [21]. To introduce HAp/NFC heterostructures into the composite material, a colloidal processing route already reported has been proposed [22]. Mechanical properties, swelling, permeability, and hydrophilicity properties were evaluated in terms of HAp content and HAp/NFC content. Finally, the biodegradation properties of the biomaterial composites were also investigated and discussed.

## 2. Materials and Methods

### 2.1. Individual Preparation and Colloidal Dispersion of HAp and NFC

Synthetic HAp with an average particle size of 3 µm (Plasma Biotal Limited, Buxton, UK) was used to formulate the colloidal suspensions involved in the preparation of materials.

The high solid content suspension of surface-modified HAp (25% vol. HAp) was formulated in deionized water along with the stabilizing agent polyethyleneimine (PEI pure < 1% water, pKa 8.6, Mw 25,000 mol/g, Sigma-Aldrich, Darmstadt, Germany) at a previously established concentration of 1.25% wt. regarding the HAp (PEI/HAp wt./wt.). Nitric acid was used to decrease the pH of this suspension to 8. To disperse the potential agglomerates, the suspension was subjected to homogenization grinding in an alumina ball mill for one hour. These modified particles are labeled as HAp1.25PEI.

NFC was obtained through a biorefinery process of an abundant agricultural lignocellulosic waste, such as wheat straw. To achieve this, cellulose fibers were extracted by an alkaline process, as previously described elsewhere [20]. Subsequently, the pretreated wheat straw was subjected to a mechanical process using a Sprout-Bauer refiner and then separated through a 0.14 mm sieving mesh. The residual lignin in the pulp was removed through a bleaching process with NaClO_2_ under acidified conditions (0.3 g of NaClO_2_ per g of fiber mixed with 2% (*v*/*v*) acetic acid) at a 3% (wt./*v*) fiber suspension for 1 h at 75 °C for three cycles. After this process, cellulose fibers were composed of 7.1 ± 0.8% extractables, 2.5 ± 0.2% lignin, 23.5 ± 0.3% hemicelluloses, 62.7 ± 3.9% α-cellulose, and 2.86 ± 0.0% ashes, as reported in a previous work [23]. Cellulose fibers were then subjected to TEMPO-mediated oxidation, as stated by Saito et al. [24], where an amount of sodium hypochlorite equivalent to an oxidation degree of 5 mmol per gram of cellulose was used. TEMPO-oxidized cellulose was subjected to a high-pressure homogenization process (HPH) using a high-pressure homogenizer (PANDA GEA 2 K NIRO, Düsseldorf, Germany) to obtain NFC. The pressure was maintained at 300, 600, and 900 bar for four, three, and three passes, respectively. The NFC obtained had the following characteristics: 1210.0 ± 15.5 µeq/g for nanofiber cationic demand, 360 ± 0.1 µeq/g for carboxyl content, 414 m^2^/g in specific surface area, 1563 nm in length, 6 nm in diameter, and 260 in the aspect ratio [20].

The dispersion and colloidal stability of both the NFC and HAp1.25PEI were initially evaluated in terms of zeta potential in the pH range of 4–11. All measurements were made by laser Doppler velocimetry in a Zetasizer Nano ZS (Malvern, Malvern, UK). The concentrations used for these assays were in the range of 0.1–0.01 g·L^−1^, using 0.01M KCl as a solvent and inert electrolyte to maintain the ionic strength of the medium. The pH values were adjusted by the addition of small volumes of 0.1 M KOH and HNO_3_ and were controlled with a pH probe (Metrohm AG, Madrid, Spain).

### 2.2. Heterostructure Formation and Colloidal Processing of the PLA-NFC/HAp Tapes

The preparation of the NFC/HAp1.25PEI heterostructures was conducted by a heterocoagulation mechanism at a specific pH value. To analyze the heterostructure interaction level and determine the maximum covering degree of HAp1.25PEI onto the NFC, a saturation curve was made by zeta potential measurements. The procedure consisted of the addition of a growing amount of HAp1.25PEI suspension on the NFC (calculated as percentage of inorganic mass over dry weight of NFC) using a mechanical homogenizer.

From this point, two families of films composed of polylactic acid (PLA 2003D with a D-isomer content of 4.25% provided by Natureworks^®^, Minnetonka, Minnetonka, MN, USA) and HAp1.25PEI/NFC heterostructures were prepared (Table 1). The polymeric matrix was previously dissolved in tetrahydrofuran (THF, Panreac, Darmstadt, Germany) at a concentration of around 80 g/L. The first family of samples, where the percentage of HAp1.25PEI with respect to the PLA varied between 10, 20, and 30 vol.% for the 10HAp10NFC, 20HAp20NFC, and 30HAp25NFC formulations, and the NFC content with respect to the HAp1.25PEI ratio was fixed at 0.33wt.%, is shown in Table 1. In this family of samples, it is important to notice that the amount of NFC with respect to the PLA varied between 0.10, 0.20, and 0.25 wt.%. In the second family of films, the content of HAp1.25PEI particles is fixed at 20 vol.% with respect to PLA, and the NFC is varying (20, 30, and 50 wt.%) with respect to the amount of HAp1.25PEI. This nomenclature is similar to saying that the second family was prepared by mixing the HAp1.25PEI suspension with ratios of 0.33wt.% NFC with respect to the HAp content, 0.42wt.% NFC, and 0.98wt.% NFC.

**Figure 1:**
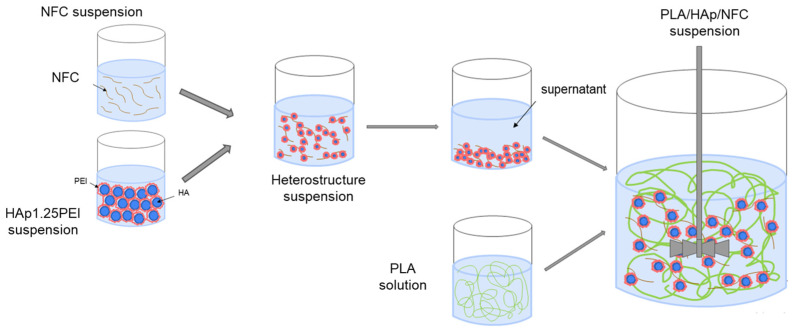
Colloidal processing of HAp and NFC suspensions.

In terms of mixture manipulation (Figure 1), all starting NFCs were diluted in deionized water and mixed with HAp1.25PEI suspension at a specific value of pH using mechanical stirring with paddles at 300 rpm for 1 h. The resulting suspensions were centrifuged and washed with THF, and then they went through a common phase of wet mixing together with the PLA solution. The final mixtures were homogenized using mechanical stirring with a disintegrator at 600 rpm for 2 h before being processed by a conventional procedure of tape casting, which was carried out at a constant cart speed of 15 mm/s, with a tape thickness of 300 µm. Once the tapes were shaped, the THF was allowed to evaporate for 24 h at room temperature before being removed.

Table 1 summarizes the main differences among the proposed formulations and the necessary amounts of each component to prepare the two families of materials.

### 2.3. Characterization of the PLA-NFC/HAp Tapes

The NFCs modified with HAp and the processed tapes were observed using a tabletop field emission scanning electron microscope TM-1000 (Hitachi, Tokyo, Japan). The tapes were prepared with carbon paint previously to the observation with a field emission scanning electron microscope (FESEM, Hitachi S4700, Japan), using a beam voltage of 10 kV. PLA-HAp/NFC composites, as well as all the starting materials, were analyzed using Attenuated Total Reflectance–Fourier Transform Infrared Spectroscopy (ATR-FTIR) using a Spectrum 3 Optica FT-IR spectrometer (Perkin Elmer, Waltham, MA, USA) equipped with an Attenuated Total Reflectance (ATR) device. The spectra were scanned in the region from 4000 to 500 cm^−1^.

The swelling index (SWI) of the tapes was determined following the protocol described by Espinosa et al. [25] with modifications. Tape samples (2 × 2 cm^2^) were weighed (S_0_) and immersed in 50 mL of distilled water with low agitation for 24 h at 25 °C. Then, a paper filter was used to remove the excess water, and the final weight of the samples was measured (S_w_). The SWI of the film samples was calculated by Equation (1).

SWI = (S_w_/S_0_) × 100 (1)

The tapes, previously immersed in distilled water for SWI determination, were dried at 105 °C for 24 h and weighed again (S_f_), and the solubility in water (SW) was calculated using Equation (2).

SW = 100 − (S_f_/S_0_ × 100) (2)

All measurements were performed in triplicate for each experimental condition, and the results are expressed as percentages (%).

The water vapor permeability (WVP) of the tapes was determined by the desiccant method according to the ASTM E96/E96M-10 standard method [26]. The tapes were placed into anodized aluminum TQC Sheen Permeability Cups (Industrial Physics, Boston, MA, USA), creating an exposed surface area of 10 cm^2^. The cups contained CaCl_2_ as desiccant material. To perform the analysis, weight measurements were taken from the capsules for 24 h. During the assay, the capsules were stored in a climatic chamber (Memmert, Büchenbach, Germany) at 25 °C and 50% RH. Water vapor transition rate (WVTR, g·h^−1^·m^−2^) and WVP (g·Pa^−1^·s^−1^·m^−2^) data were obtained using Equations (3) and (4), respectively.

WVTR = G/tA(3)

WVP = WVTR/∆p = WVTR/S (R1 − R2)(4)

where G is the weight change (g), t is the time during which G occurred (h), A is the test area (cup mouth area, m^2^), ∆p is the vapor pressure difference (Pa), S is the saturation vapor pressure at test temperature (Pa), and R1 and R2 are the relative humidities at the source at the vapor sink, respectively, expressed as a fraction. All measurements were performed in triplicate, and the results are expressed as the arithmetic mean with its standard deviation.

Tape mechanical properties in terms of tensile strength, Young’s modulus, and strain–stress curves were measured according to the ASTM D882 method [27]. For that, the tapes were cut into rectangular strips of 10 cm in gauge length and 1.5 cm wide and evaluated using a Universal Testing Machine model LF Plus Lloyd Instrument AMETEK Measurement & Calibration Technologies Division (Largo, FL, USA) of a 1 kN load cell. The initial grip separation and crosshead speed were set at 65 mm and 10 mm/min, respectively. Results are expressed as an average of six samples for each type of tape.

All data are represented as the average ± standard deviation. Data were analyzed by analysis of variance (ANOVA) followed by the Duncan test. Statistical analyses were performed with SPSS software (version 30.0.0) and are indicated as different letters that show significant differences (*p* ≤ 0.05).

## 3. Results and Discussion

### 3.1. Surface Modification of Involved Materials and Heterostructure Formation

The main challenge presented by cellulose processing is its hygroscopic behavior, which hinders its dispersion in the hydrophobic PLA matrix. The mixture of HAp inorganic particles with NFC creates a heterostructure under a colloidal approach, and its distribution, assembly, and immobilization control provokes a reduction in heterogeneities within the final structures of the shaped materials.

**Figure 2:**
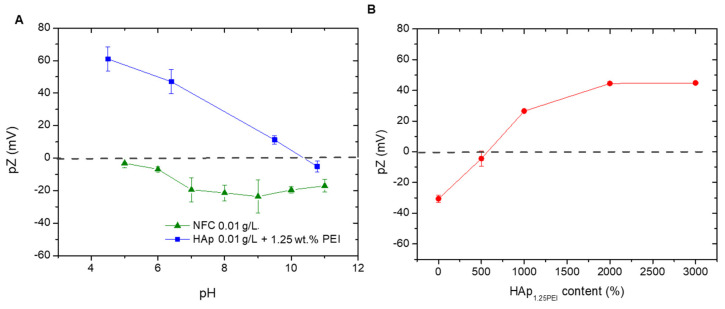
Zeta potential evolution as a function of the pH media (**A**) and its evolution as a function of the weight of HAp1.25PEI added on the NFC surface (**B**).

In order to determine the optimum colloidal conditions to create the HAp/NFC heterostructure, both components were evaluated individually by zeta potential. Previously, the adsorption of 1.25 wt.% of branched PEI on the HAp particles’ surface was carried out in deionized water at a pH of 8 in order to stabilize this phase and create HAp1.25PEI particles, following a previously described methodology [21]. After that, the evolution of the zeta potential of HAp1.25PEI and NFC was examined in a wide range of pH values (pH 4–11) (Figure 2A). These measurements allow selecting the appropriate pH conditions that ensure an effective electrostatic interaction between the NFC and the modified HAp particles in the heterostructure. As expected, the surfaces of NFC were negative for the mentioned pH range. The surface charge of the nanofibers conferred by the hydroxyl and carboxyl groups resulted in values close to −20 and −25 mV. The repulsive electrostatic forces favor the stability of the fibers in the medium and, therefore, their suitability and efficiency as a structural matrix in the production of composite tapes. On the other hand, the surface charge of the modified HAp1.25PEI particles was positive over almost the total pH range, achieving values around 60–30 mV. Above a pH of 7–8, their zeta potential values presented a less positive charge as a consequence of the conformational changes in the polyelectrolyte chains and the possible dissolution of some cations of the HAp at a more extreme basic pH.

In order to create the heterostructure, the best interaction of both components is promoted by combining the highest positive charge of the amine groups of PEI with the largest negative charge of the hydroxyl/carboxyl groups of the NFC. This procedure has already been reported elsewhere but using other different inorganic species [20,21]. According to the surface charge balances of both species, the working pH for formulating the composite mixtures was around 7. At these pH conditions, the electrostatic attraction between both compounds is considered adequate to build the heterostructure.

To set the quantity of HAp that fully covers and modifies the particle surface, different concentrations were added (referring NFC content). Figure 2B displays the variation in the NFC zeta potential with the addition of HAp1.25PEI and the formation of the heterostructure at a pH of 7. The positively charged HAp1.25PEI approaches the negative surfaces of NFC, firstly producing the neutralization of its functional groups. Then, the negative zeta potential values of bare NFC shifted towards a value of 30 mV when the percentage of the modified HAp was 1000 wt.%. After that, the values continued increasing until a maximum of around 45 mV, when the added percentage was 2000 wt.%. No further variations in terms of surface charge were observed for higher additions of the inorganic phase.

**Figure 3:**
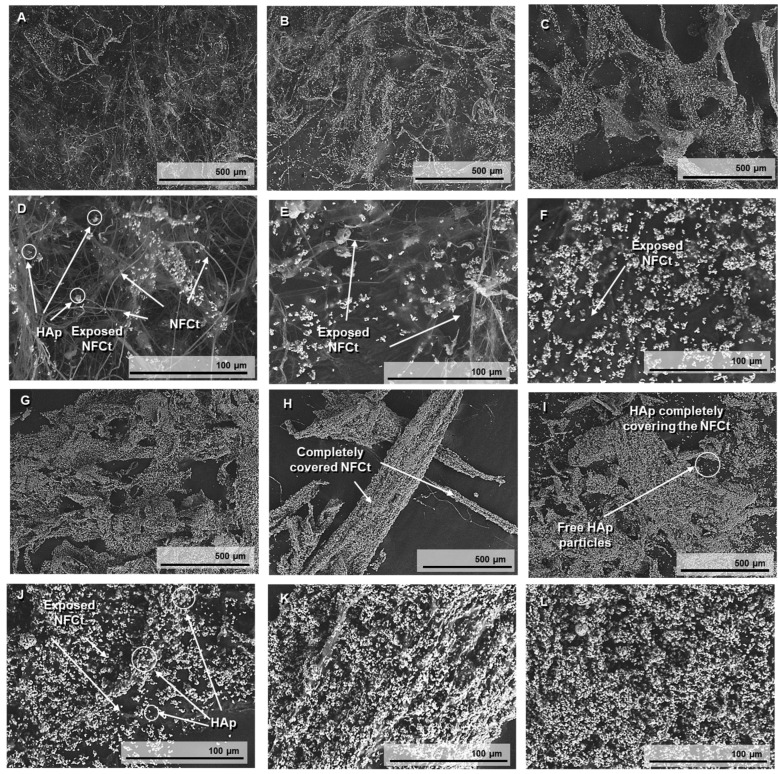
SEM images taken at 150× and 1000× magnification of lyophilized and surface-modified NFC with 100 %wt. HAp (**A**,**D**), 200 wt.% HA (**B**,**E**), 500 wt.% HAp (**C**,**F**), 1000 wt.% HAp (**G**,**J**), 2000 wt.% HAp (**H**,**K**), and 3000 wt.% HAp (**I**,**L**).

The SEM images in Figure 3 are useful to prove the surface modification of the NFC with the different concentrations of HAp1.25PEI adsorbed. HAp particles can be distinguished as bright white dots in the images, while the NFC appears as a network of fibers on which HAp is deposited. In the images referred to as <500 wt.% of HAp1.25PEI (Figure 3A–D), the fibrous nanostructures of the NFC are partially coated. As the HAp percentage increases to 500% and 1000% (Figure 3E–H), the presence of HAp1.25PEI becomes more apparent, but exposed NFC surfaces are still visible. However, at higher percentages of 2000% and 3000 wt.% of HAp1.25PEI (Figure 3I–L), empty surfaces are no longer visible, and only the agglomerates of NFC coated with modified HAp particles can be distinguished. Between the percentages of 2000% and 3000%, there is a notable difference. At 3000 wt.%, free HAp particles can be observed, which confirms surface saturation at the value of 2000%. This also indicates that at higher concentrations, the HAp particles cease to anchor to the NFC and instead remain free in the suspension.

### 3.2. Evaluation of Biomaterials with Different HAp Contents

**Figure 4:**
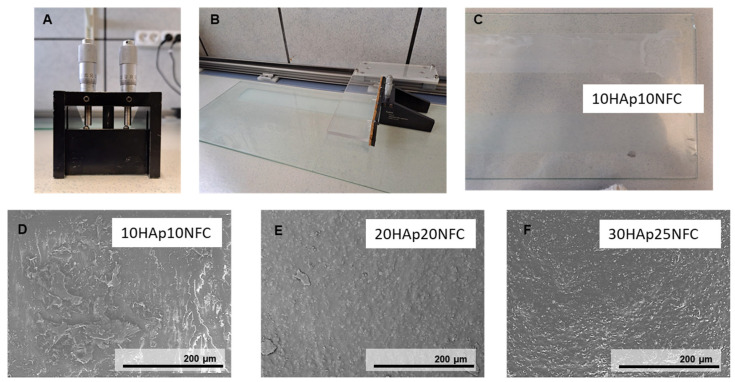
Tape casting process for the PLA/HAp/NFC composites consisting of doctor blade apparatus (**A**), pushed by an engine on a surface (**B**) where the films are dried (**C**). Micrographies of 10HAp10NFC (**D**), 20HAp20NFC (**E**), and 30HAp25NFC (**F**) composite tapes.

After determining the maximum amount of HAp necessary to stabilize the NFC, we determined the total amount of HA required to have an effect on the biodegradation of the biomaterial according to the literature. According to the literature, 30 and 80 wt.% HAp is required in polymer-based composites to obtain highly bioactive composites [28]. An amount of 20 vol% has already been tested by the research group, obtaining a good relation between cell viability and biodegradation. Consequently, the first family of three composites (10, 20, 30 vol.%, named 10HAp10NFC, 20HAp20NFC, and 30HAp25NFC, respectively) where the ratio of HAp/NFC is fixed, was analyzed in order to determine the adequate amount of ceramic phase to later define the amount of NFC necessary to create a high and customized porosity. In this first analysis, the content of NFC was fixed in concordance with the amount of HAp in a ratio of 0.33 wt.% of NFC/HAp. Figure 4A–C show the doctor blade used to process the composite tapes. The SEM micrographs for the first family of composite tapes, 10HAp10NFC (Figure 4D), 20HAp20NFC (Figure 4E), and 30HAp25NFC (Figure 4F), are also shown in Figure 4. Both showed similar surfaces with an increased roughness with the HAp/NFC content increase.

Many studies in the literature report that the addition of HAp to PLA matrices leads to a notable decrease in the mechanical strength of the matrices. This decrease is usually associated with dispersion issues due to the formation of agglomerates when using unmodified HAp [29]. The use of surface-modified Hap, with PEI as a stabilizing agent, as described in the present work, aims to overcome these limitations. The success of this method, based on the application of colloidal chemistry protocols, has been previously reported in the research group [11,30]. However, the addition of new phases to the PLA matrix, such as HAp/NFC complexes, remains a challenge to pay attention to in the investigation of new materials. The incorporation of NFC into polymeric matrices usually leads to a considerable increase in the mechanical performance of composites due to the inherent stiffness of NFC chains through intermolecular and intramolecular hydrogen bonds, their homogeneous distribution in the matrix, and high compatibility with the polymeric matrix through high hydrogen-bonding interaction. These effects are observed up to a certain amount of NFC content, since excessive content leads to the clustering of the nanofibers in the matrix, behaving as weak points that initiate the final failure of the composite [25].

**Figure 5:**
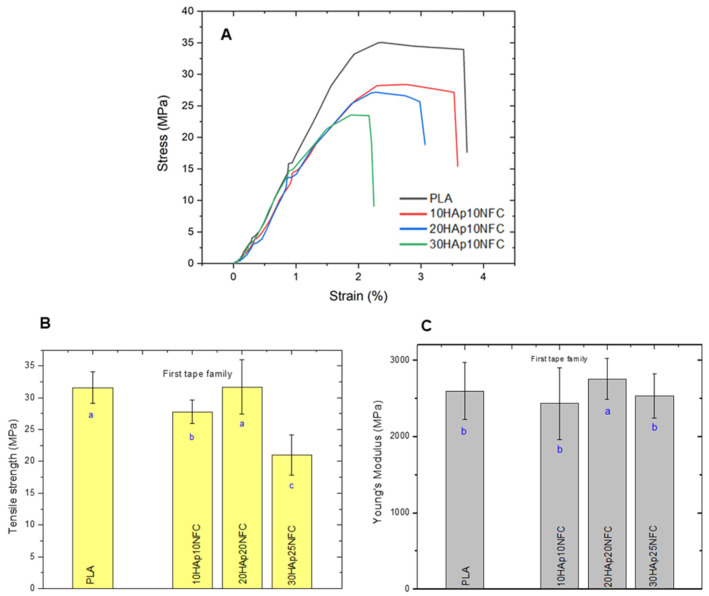
Mechanical properties of PLA, 10HAp10NFC, 20HAp20NFC, and 30HAp25NFC tapes in terms of strain–stress curves (**A**), tensile strength (**B**), and Young’s modulus (**C**). Lowercase (a, b and c) letters show significant differences (*p* ≤ 0.05).

Therefore, the mechanical properties of the first family of tapes with different contents of HAp and a fixed amount of HAp/NFC were determined (Figure 5). As expected, the stress–strain curves of the tapes showed that the presence of the heterostructure (HAp/NFC complexes) made the materials slightly more brittle. However, the presence of NFC seems to overcome the detrimental effect that the presence of HAp could have. Thus, it was observed that, at best, there were no significant differences between the tensile strength of 20HAp20NFC (31.68 MPa) and PLA (31.58 MPa). A similar effect was found for Young’s modulus. It has been reported in the literature that the mechanical properties of PLA-HAp composites are highly dependent on the dispersion of HAp in the PLA matrix. It appears that the presence of the fixed ratio of 0.33 wt.% NFC/HAp was optimal to achieve this correct dispersion and hence the maintenance of the mechanical performance. Low amounts of the HAp/NFC complex (tape 10HAp/10NFC) significantly decrease the tensile strength of PLA. These low amounts of HAp/NFC can cause micro-agglomerations, resulting in poor compatibility of the complex with PLA and difficulty in evenly dispersing the HAp/NFC, leading to a decrease in the tensile strength of the tapes. When the ratio is increased (tape 20HAp/20NFC), it seems that the adhesion of the complex to the PLA is stronger, since the NFC can withstand high stresses. The PLA matrix is prone to viscoelastic deformation and plastic flow, so the composite tape exhibits transmission stress. The addition of higher amounts (tape 30HAp25NFC) caused a decrease in the mechanical performance. The high concentration of the complex in the matrix could be causing the formation of microstructural defects/obstacles that act as weak breaking points. Similar effects have been reported for PLA films incorporating stearic acid-modified nanocellulose [31].

**Figure 6:**
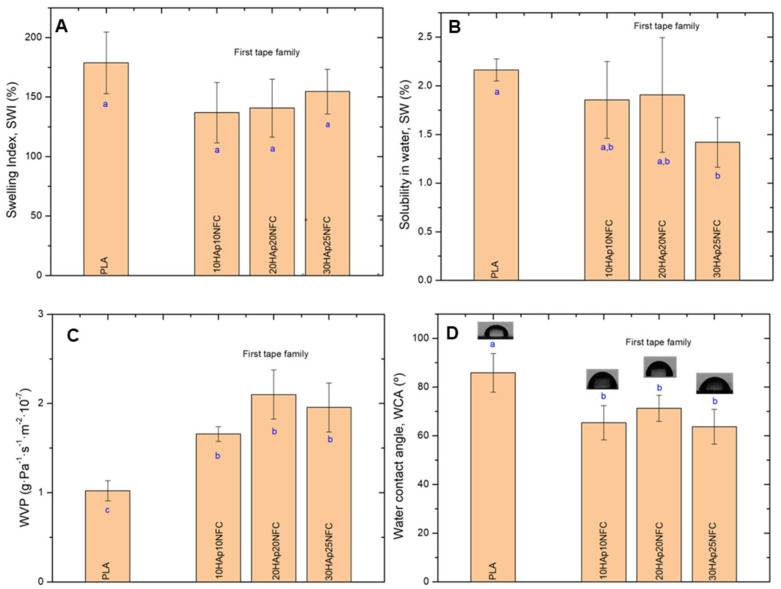
Physical properties of the first family tapes: swelling index (SWI, (**A**)), solubility in water (SW, (**B**)), water vapor permeability (WVP, (**C**)), and water contact angle (WCA, (**D**)). Lowercase (a, b and c) letters show significant differences (*p* ≤ 0.05).

HAp/NFC complexes’ effects on PLA-based composite tape physical properties were also examined (Figure 6). It was observed that the presence of HAp/NFC complexes on PLA matrices did not significantly change the SWI of the neat PLA (178.90%, Figure 6A). Although PLA is hydrophobic, the weight gain of the tapes due to water absorption is usually attributed to the hydrophilic nature of the ester groups in PLA [32]. The SW of the composite tapes was very low (less than 2.5%), as reported in the literature for PLA-based films [33]. It was observed that the presence of HAp/NFC complexes led to a mild decrease in the SW (Figure 6B). The strong adhesion between the PLA and HAp/NFC complexes, suggested by the mechanical performance of the tapes, could be the reason for this drop. WVP was the most affected property. Thus, the hydrophobic nature of PLA was demonstrated by a low WVP value (1.02 g·Pa^−1^·s^−1^·m^−2^·10^−7^). When HAp/NFC was incorporated into the first family of tapes formed, the permeability of the tapes was found to increase by about 50% (Figure 6C). This increase was independent of the amount of complex included in the formulation, but it was slightly higher for the 20HAp20NFC composite. Generally, the WVP in manufactured layers is strongly influenced by the hydrophilic or hydrophobic nature of the materials, the manufacturing process, the type, amount, and distribution of additives applied, the presence of voids, and the final arrangement of the polymeric structure. Similar results to the present were reported by Abdulkhani et al., finding an increase in the WVP of PLA films as the addition of acetylated NFC increased [34]. The hydrophilic nature of the HAp microparticles and the NFC chains could be the cause of this increase. According to the microstructure analysis (Figure 4), the HAp/NFC composite tapes showed higher roughness, which could be related to a structure with more voids that promotes water vapor permeation. Therefore, it could be said that there is a synergistic effect between HAp and NFC on the moisture penetrability of the tapes. Finally, the increase in the hydrophilicity of the tapes was observed by measuring the water contact angle (WCA). In all cases, the inclusion of the HAp/NFC complex resulted in a significant decrease in WCA values with respect to neat PLA (Figure 6D).

### 3.3. Evaluation of Biomaterials with Different NFC Contents

Considering the above characterization, the optimal amount of HAp was determined to be 20 vol.%, which is able to counter the mechanical properties’ decrease associated with the HAp/NFC incorporation, and that presents better swelling, permeability, and hydrophilicity properties compared with the 10 and 30 vol% of HAp. The ratio of the NFC of this composite was 0.2 wt.%. Since the NFC content may influence the porosity and degradation of biomaterials, further characterization was performed on the second tape family, where the HAp was fixed at 20 vol.% and the NFC content varied (20HAp20NFC, 20HAp30NFC, and 20HAp50NFC).

**Figure 7:**
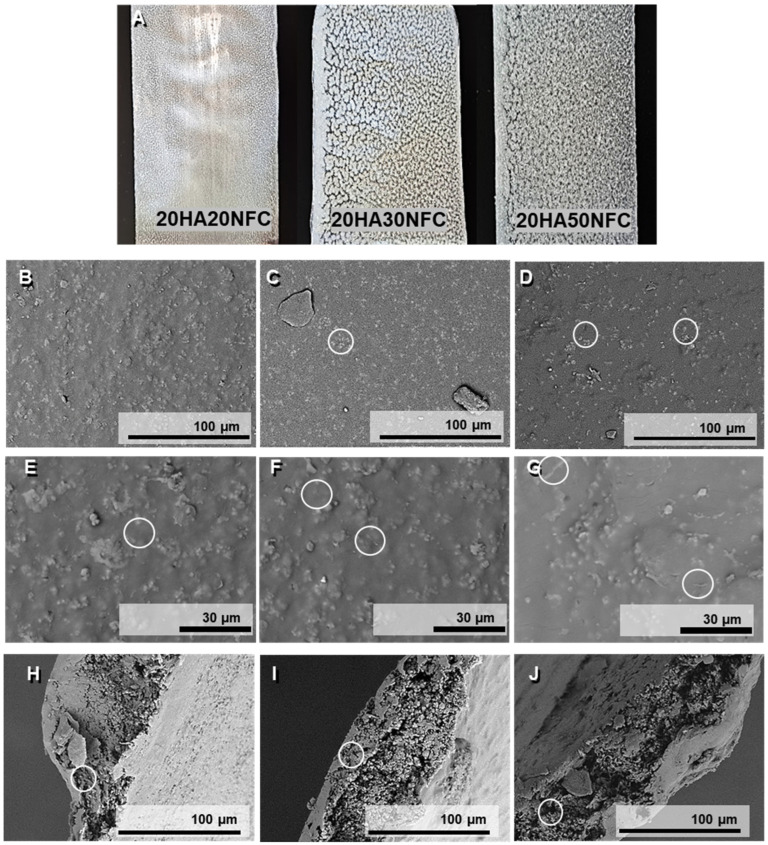
Tape macroscopic images (**A**). SEM images of the surface, and cross-section of the tapes of 20HAp20NFC (**B**,**E**,**H**), 20HAp30NFC (**C**,**F**,**I**), and 20HAp50NFC (**D**,**G**,**J**) with HAp and NFC highlithed in white circles.

In Figure 7, the macroscopic images of the tapes are shown. The tapes with a lower NFC content exhibit a more homogeneous appearance without visible agglomerates, as opposed to the tapes with higher concentrations, which are rougher in texture. Figure 7 shows the micrographs of the surface (Figure 7B–G) and the cross-section of the tapes (Figure 7H–J) of the three processed compositions. A mostly homogeneous surface can be observed, where the HAp (Figure 7C,D) and NFC (Figure 7E–G) are visible and dispersed along the tape surfaces. However, several clusters can be seen on the surface, which could be explained by the anchoring of multiple HAp particles to each nanofiber, leading to structural agglomeration since the NFCs cannot be distinguished due to their inclusion in the polymer matrix. The homogeneity is confirmed, and no cracks are evident in the material. In the cross-section of the samples, the composition’s structure can be discerned as an amorphous PLA matrix with embedded white HAp particles. An internal porosity formed by small-sized pores can also be distinguished, and this internal porosity is more evident with the increasing amount of NFC [35].

**Figure 8:**
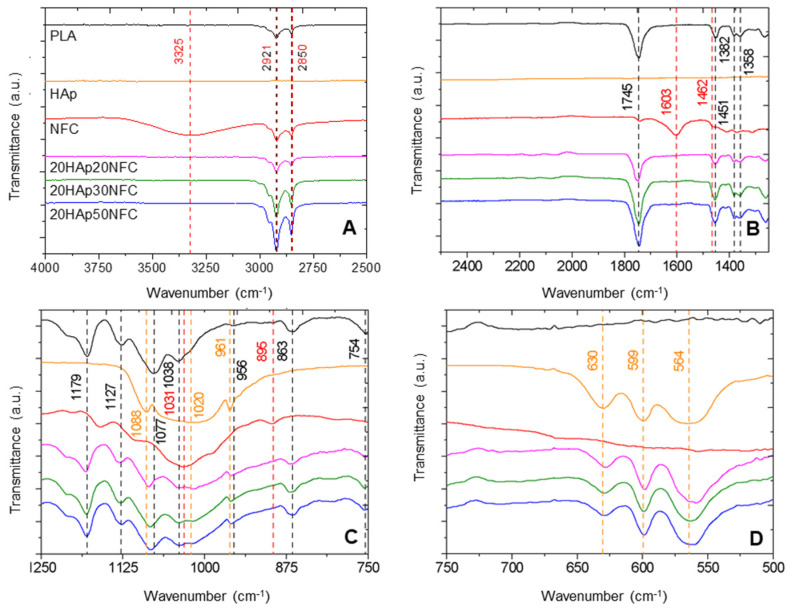
FTIR in the wavenumber ranges of 4000–2500 cm^−1^ (**A**), 2500–1250 cm^−1^ (**B**), 1250–750 cm^−1^ (**C**), and 750–500 cm^−1^ (**D**) of PLA, Hap, and NFC and 20HAp20NFC, 20HAp30NFC, and 20HAp50. Each band is identified with dashed lines, and its wavenumber is shown in the color of the component that generates it. In the case of overlapping peaks from multiple components, their dashed lines and wavenumbers are represented with alternating colors. In the case of overlapping peaks from multiple components, their dashed lines and wavenumbers are represented with alternating colors (black PLA, red NFC, yellow HAp).

The next step was to analyze the possible interactions that occur between the different phases present (PLA, HAp, and NFC), as well as any modifications that may have occurred during processing. Fourier Transform Infrared (FTIR) Spectroscopy (Figure 8) was used to determine the changes introduced in the structure by comparing the spectra of the composite materials with those of the starting materials.

In Figure 8, the spectra of the three studied compositions and their precursors are shown. Each band is identified with dashed lines, and its wavenumber is shown in the color of the component that generates it. In the composites, the most characteristic bands of PLA can be observed, such as 2850 cm^−1^ of the CH_2_ stretching, 1382 cm^−1^ attributed to the CH_3_ group, or 1451 cm^−1^ for the symmetric bending of CH_3_ [36]. Similarly, the bands associated with HAp, such as 564 cm^−1^, 599 cm^−1^, 1020 cm^−1^, and 1088 cm^−1^, associated with the phosphate group (PO_4_^3−^) vibrations, can be found in the composites [37]. In the spectra, the characteristic bands of NFC mostly disappear due to its low concentration. However, close to the HAp band at 1020 cm^−1^, a broader peak appears for NFC at 1031 cm^−1^. Other bands can be correlated to PLA and NFC, such as those detected at 2921 cm^−1^ and 2850 cm^−1^ of the CH stretching of NFC and the CH_2_ asymmetric and symmetric bending of PLA, and 1745 cm^−1^ of the C = O stretching band of PLA, growing in intensity with the NFC addition. This increase is associated with the strong interaction in the heterostructure of HAp/NFC and of this structure with the PLA polymer matrix [38]. This explanation is also reflected in the most characteristic PLA band at 1745 cm^−1^, which is highly sensitive to the changes caused by the interactions with other components, and in this case, increases noticeably with the NFC content, as observed in the spectra of the different composite samples.

Therefore, it can be concluded that after the mixing processes in solution/suspension and subsequent tape casting, the composite materials do not undergo degradation or alterations in their crystalline structure. However, they do ensure a strong bond between the matrix and the heterostructure (NFC and HAp).

**Figure 9:**
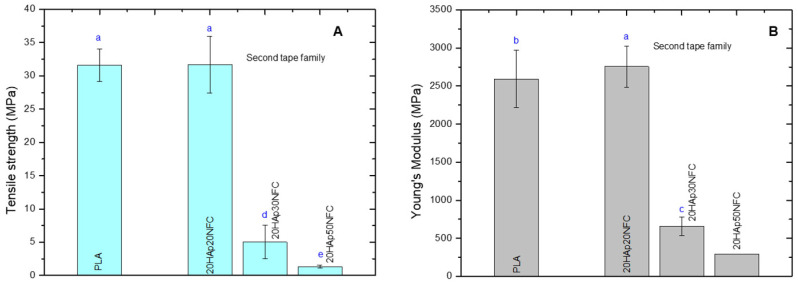
Mechanical properties of the PLA, 20HAp20NFC, 20HAp30NFC, and 20HAp50NFC tapes in terms tensile strength (**A**), and Young’s modulus (**B**). Lowercase (a, b, c, d and e) letters show significant differences (*p* ≤ 0.05).

After that, the mechanical properties of the composites were analyzed similarly to the first family in order to observe the modification associated with NFC phase increases (Figure 9).

After setting the proportion of the HAp/NFC complex at 20%, a second family of tapes was prepared, in which the amount of NFC was increased. As expected from the results obtained in the first family, the presence of higher proportions of NFC causes a drastic drop in the mechanical property values. These results confirm that the proportion of the HAp/NFC and the amount of heterostructure in the composite directly interfere negatively with the mechanical properties. This behavior is associated with the increase in porosity in the microstructure of the tapes. The proportion of 0.33 wt.% of HAp/NFC of 20HAp20NFC presents the better mechanical properties because it is the composite with less porosity compare with 30HAp30NFC and 20HAp50NFC. Even though the reinforcement of NFC improves the mechanical properties of the composite, as was observed in the first family of composites, the high increase in the NFC and heterogeneous porosity that it created may be responsible for the loss of the mechanical properties in terms of tensile strength and Young’s modulus [39]. 

**Figure 10:**
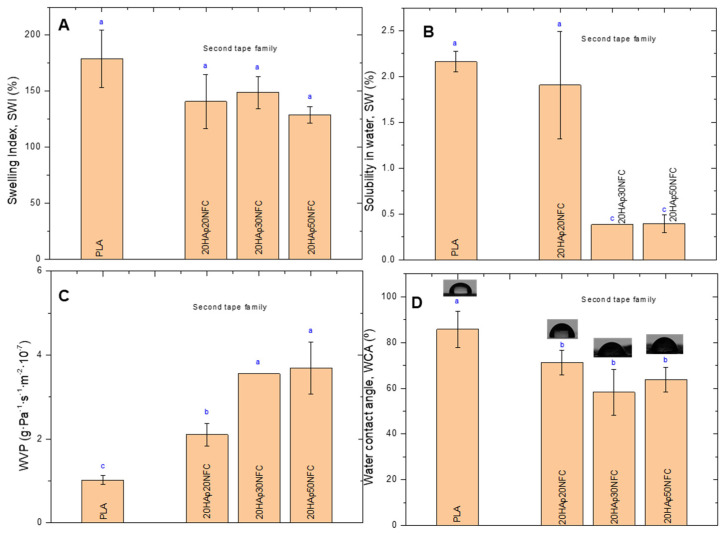
Physical properties of the tapes: swelling index (SWI, (**A**)), solubility in water (SW, (**B**)), water vapor permeability (WVP, (**C**)), and water contact angle (WCA, (**D**)). Lowercase (a, b and c) letters show significant differences (*p* ≤ 0.05).

The second family’s physical properties were also examined (Figure 10). Similarly, to the first family, the increase in NFC on PLA matrices does not significantly change the SWI of the neat PLA. However, the solubility decreases noticeably with the increase in the NFC in 20HAp30NFC and 20HAp50NFC. This may be associated with the increase in the ratio of NFC/HAp that masks the hydrophilicity of HAp microparticles in the heterostructure. A higher proportion of NFC in the tapes led to a nearly fourfold increase in WVP (Figure 10C). As suggested in the mechanical properties evaluation, the presence of high concentrations of NFC in the structure causes its disruption. Agglomerates are formed, which, in addition to giving rise to break points, result in tapes with a very heterogeneous microstructure through which water vapor molecules diffuse more freely, increasing the permeability of the materials. Finally, the increase in the hydrophilicity of the tapes was observed by measuring the WCA, where the increase in NFC resulted in a significant decrease in the WCA values with respect to neat PLA (Figure 10D).

### 3.4. Degradation Characterization

Once the porosity has been determined, the next objective is to evaluate how this property is able to modify and therefore improve the degradation rate of these biomaterials. The porosity in the material increases its specific surface so that, as the material degrades inside the patient, the medium can penetrate the material through its pores, increasing the surface in contact with it and, therefore, its degradation rate [40]. During the PLA degradation, the matrix porosity increases, which exposes the HAp particles. The HAp degradation process produces a release of calcium ions that play a crucial role in the bone regeneration process.

After producing samples with different concentrations of NFC, tests were carried out to characterize the two factors of its degradation: water absorption and mass loss. These parameters and, therefore, the biodegradability of the composite materials will be evaluated in the tape-shaped materials. The tapes of the three concentrations were immersed in a PBS buffer solution widely used to imitate the environment to which the material will be subjected inside the patient, as it has a concentration of ions very similar to that of the extracellular fluid of mammals [41]. The pH was monitored during the experiment, and no significant changes were observed in the PBS medium.

**Figure 11:**
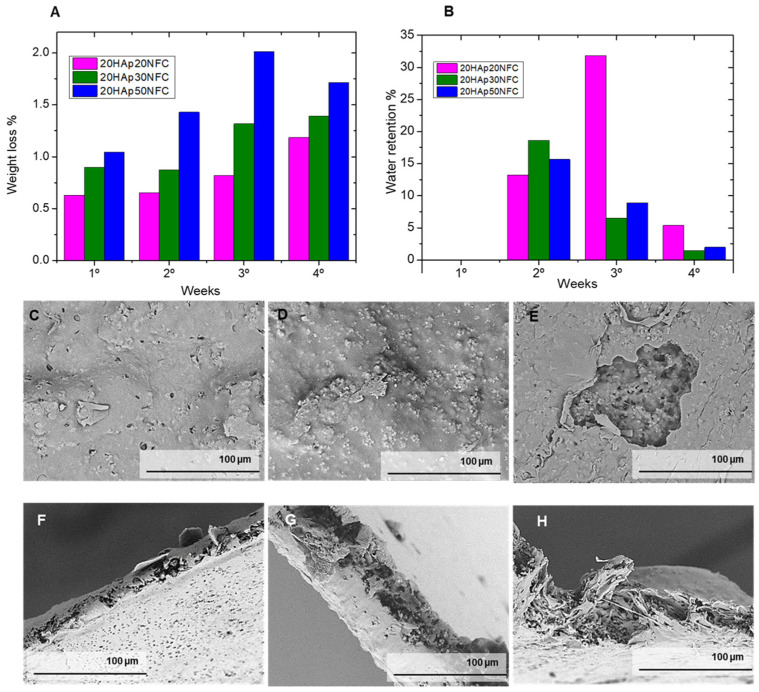
Weight loss (%, (**A**)) and water retention (%, (**B**)) of samples in a 4-week degradation study. After the degradation tests, the tape samples were analyzed by a scanning electron microscope (**C**–**H**) to observe the changes in their structure.

Figure 11A shows the graph of the evolution of the cumulative weight loss over four weeks, represented as a percentage. It can be determined that, through the experiment, the samples have a constant loss of mass. Furthermore, it can be verified that there is a correlation between this and the concentration of NFC in the sample. The composition with a higher concentration of NFC in the matrix (20HAp50NFC) shows the highest percentage of loss of the three compositions at 1.71%, the 20HAp30NFC of 1.40%, while the tape with a lower concentration of NFC 20HAp20NFC loses a lower mass fraction of 1.18%. These values of weight loss are in the low range of other reported PLA/HAp scaffolds, biomaterials, and films composites [42], which could be associated with the low thickness of the evaluated composites (300 µm), since the greater the thickness of the devices, the faster the degradation [43]. This factor is directly related to the fact that a higher concentration of cellulose is expected to have a greater porosity and, therefore, a greater number of potential degradation centers in the material. Therefore, thanks to the gradual addition of cellulose, the degradation rate of the compounds can be modified in the first days of implantation.

Figure 11B represents the evolution of the water absorption of the samples during the test. This is an experiment that generally follows a less clear trend compared to weight loss tests. This is mainly due to the fact that water adsorption is usually related to two factors: as the sample degrades it loses mass, so the amount of water absorption is lower, but as the sample degrades, a greater number of porosities and cavities capable of holding more medium provide a higher adsorption value. In the first week, the initial degradation observed in the mass loss tests was perfectly compensated by the adsorption of water, giving practically negative values. In the second week, the porosity generated by degradation allowed an increase in water adsorption, and the two behaviors could be observed. At high cellulose contents, mass loss begins to be observed preferentially, but at low cellulose contents, where the degradation is not yet so high, a significant increase in water adsorption occurs. Finally, in the fourth week, the degradation and the water absorption are again balanced.

Therefore, from these tests, it can be concluded that the addition of nanocellulose to the compounds not only modifies the degradation but also the hydrophilicity of the compounds. This modification has been proved with low NFC amounts from 0.2 to 0.5 wt.%, obtaining a variation in the weight loss proportion from 1.18 to 1.71%, respectively, and in the contact angle from 71° to 64°. These are crucial parameters for future cell culture in vitro and in vivo experiments and for the biocompatibility of future implants.

In Figure 11C–H, it can be observed how porosity has been created on the surface of the tapes caused by the degradation of the PLA that has left the HAp exposed, part of it dissolving in the buffer solution, leaving its empty hole in the matrix. In the case of the composition with a higher concentration of NFC, it can be seen how larger voids have emerged, which confirms the hypothesis that the higher the concentration of NFC, the greater the porosity that is generated and the higher the degradation rate. In the case of the cross-section of the tapes, the exposed HAp particles also decreased due to their dissolution in the PBS, leaving a greater proportion of the polymer matrix. These observations agree with the results obtained in the degradation tests, where, by immersing the tapes in a buffer solution, the natural degradation that would take place inside the patient’s body is recreated. In this, the implant suffers a loss of mass due to the combination of the degradation of the polymer matrix and the dissolution of the HAp in the medium.

## 4. Conclusions

This study aimed to create a new biodegradable biomaterial by combining cellulose nanofibers with a composite of hydroxyapatite and polylactic acid. The goal was to control porosity to obtain a gradient of biodegradation profiles in the composite family. The initial step involved modifying the cellulose nanofibers’ surface to ensure their integration into the hydrophobic PLA matrix. This modification was achieved using hydroxyapatite (HAp), enhancing interaction between the particles and creating heterostructures of HAp/NFC. Zeta potential analysis identified a pH of 8 as the optimal pH for stable bonding between HAp and NFC, and SEM images confirmed the successful anchoring of HAp nanoparticles to the cellulose nanofibers’ surface.

A first family of composites was processed in tape shape (10HAp10NFC, 20HAp20NFC, and 30HAp25NFC, respectively), where the ratio of HAp/NFC was fixed in order to determine the adequate amount of ceramic phase to later define the amount of NFC necessary to create a high and customized porosity. The optimal amount of HA was 20 vol.%, which is able to counter the mechanical property decrease associated with the HAp/NFC incorporation and that presents better swelling, permeability, and hydrophilicity properties. A second family of composites, where the HA was fixed at 20 vol.% and the NFC content varies (20HAp20NFC, 20HAp30NFC, and 20HAp50NFC), was then analyzed. Even with the loss of mechanical properties in terms of tensile strength, Young’s modulus, and the slight modification in the hydrophilicity and permeability, the biodegradation properties are highly influenced by the NFC increase in the composite. Degradation studies highlighted the direct relationship between NFC concentration and material degradation rates within a simulated physiological environment.

In summary, this investigation effectively created a biocompatible and biodegradable porous biomaterial. The integration of cellulose nanofibers into a hydroxyapatite–polylactic acid composite resulted in controlled porosity and, therefore, in the capacity to personalize degradation rates through NFC content modulation, thus enabling tailored material attributes according to patient requirements.

## Figures and Tables

**Table 1:** Composition of the initial suspension and the final composites after the drying of the two families of evaluated materials.

	Initial Aqueous Suspension			Final Composites * All Proportions of the Final Composites Are Related to PLA Content.			
1st family of samples	% vol. HAp/H_2_O	wt.% NFC * related HAp	wt.% HAp * related NFC	% vol. HAp	% wt. cellulose	% vol. HAp-NFC	% free HAp
30HAp25NFC	25%	0.33	30,000	30%	0.25	2.00	27.9
20HAp20NFC	25%	0.33	30,000	20%	0.20	1.32	18.65
10HAp10NFC	25%	0.33	30,000	10%	0.10	0.65	9.33
2nd family of samples	% vol. HAp/H_2_O	wt.% NFC * related HAp	wt.% HAp * related NFC	% vol. HAp	% wt. cellulose	% vol. HAp-NFC	% free HAp
20HAp20NFC	25%	0.33	30,000	20%	0.20	1.32	18.65
20HAp30NFC	25%	0.42	24,000	20%	0.30	2.00	19.18
20HAp50NFC	25%	0.98	10,000	20%	0.50	4.16	17.10

## Data Availability

The original contributions presented in this study are included in the article. Further inquiries can be directed to the corresponding author.
